# Effectiveness of Combined Whole-Body Vibration and Intensive Therapeutic Exercise on Functional Capacity in Children with Cerebral Palsy: A Randomized Controlled Trial

**DOI:** 10.3390/medicina61050873

**Published:** 2025-05-09

**Authors:** Iñigo Monzón-Tobalina, Rosa María Ortiz-Gutiérrez, Ángela Concepción Álvarez-Melcón, Álvaro Pérez-Somarriba, Patricia Martín-Casas, María José Díaz-Arribas

**Affiliations:** 1 Doctoral Program in Healthcare, Faculty of Nursing, Physiotherapy and Podiatry, Universidad Complutense de Madrid, 28040 Madrid, Spain; inmonzon@ucm.es; 2 Physical Therapy and Rehabilitation Unit, Hospital Infantil Universitario Niño Jesús, 28007 Madrid, Spain; aperezso@ucm.es; 3 Department of Radiology, Rehabilitation and Physiotherapy, Faculty of Nursing, Physiotherapy and Podiatry, Universidad Complutense de Madrid, 28040 Madrid, Spain; rosaorti@ucm.es (R.M.O.-G.); angela.alvarez@ucm.es (Á.C.Á.-M.); mjdiazar@med.ucm.es (M.J.D.-A.); 4 InPhysio Research Group, Health Research Institute of the Hospital Clínico San Carlos (IdISSC), 28040 Madrid, Spain; 5 Comprehensive Integral Rehabilitation, Physiotherapy and Neurorehabilitation Research Group, Universidad Complutense de Madrid, 28040 Madrid, Spain

**Keywords:** spastic cerebral palsy, whole-body vibration, physical therapy, gross motor function, lower limb

## Abstract

*Background and Objectives:* Whole-body vibration (WBV) therapy presents controversial evidence regarding its effectiveness in improving lower limb functional capacity in children with cerebral palsy (CP), particularly when applied continuously as an adjunct to a physiotherapy program with demonstrated efficacy. This study aimed to evaluate the effectiveness of adding WBV to an intensive therapeutic exercise and functional training program in improving lower limb functional capacity in children with spastic CP. *Materials and Methods:* Thirty children with spastic CP were randomly assigned to a control or experimental group. Both groups completed a 4-week intensive therapeutic exercise and functional training program (4 sessions/week). The experimental group additionally received daily WBV. *Results:* Both groups showed significant improvements in all analysed variables at 1, 2, and 6 months post-treatment (*p* < 0.001). However, no significant between-group differences were found for primary (GMFM-88 D: *p* = 0.80; GMFM-88 E: *p* = 0.91) or secondary outcomes in relation to muscle tone and strength, and balance. A trend toward greater improvement was observed in the experimental group but without statistical significance. *Conclusions:* The addition of WBV to an intensive program of therapeutic exercise and functional training does not yield additional benefits in motor function, spasticity, gait capacity, lower limb muscle strength, or balance compared to intensive physiotherapy and functional training alone in children with spastic CP. The significant within-group improvements can be attributed to the intensive physiotherapy intervention, comprising therapeutic exercises and functional training.

## 1. Introduction

Cerebral palsy (CP) is a non-progressive neurological disorder characterized by movement and postural dysfunctions, with an incidence of 1.5–3 per 1000 live births. Spastic CP is the most prevalent form [1,2], often presenting as diplegia [3,4]. It results from pyramidal system damage, leading to abnormal motor development, increased muscle tone, hyperreflexia, weakness, and balance issues, which can significantly affect independence, social participation, and quality of life, including sexual health [5].

The severity of motor dysfunction correlates with the extent of brain damage [6] and is commonly classified using the Gross Motor Function Classification System (GMFCS), a five-level scale where higher levels indicate greater impairment [7].

Conservative treatments aim to improve motor function and regulate muscle tone through pharmacological and physiotherapeutic interventions. Physiotherapy approaches include therapeutic exercise, functional training, and techniques such as Bobath Concept, Vojta Therapy, Proprioceptive Neuromuscular Facilitation, electrostimulation, and the use of orthotic suits. However, not all these methods are supported by strong scientific evidence, particularly regarding long-term outcomes [8,9,10,11]. In contrast, interventions focusing on goal-directed activities and muscle strengthening have demonstrated benefits in physical condition, activity levels, mobility, participation, and overall quality of life [12,13,14,15].

Whole-body vibration (WBV) has emerged as a potential adjunct therapy for CP. By generating mechanical oscillations, WBV is believed to activate muscle spindles and modulate the neuromuscular system at the spinal level, thereby reducing exaggerated stretch reflexes and promoting motor unit recruitment [16,17]. These effects may contribute to reduced spasticity, improved muscle tone regulation, and enhanced sensorimotor integration. Preliminary studies suggest that WBV may improve coordination, increase muscle strength, and enhance joint range of motion in individuals with CP [16,18].

Recent systematic reviews and meta-analyses indicate that WBV, when applied alone or in combination with other interventions, can yield functional benefits in children with CP [19,20]. However, the existing evidence is limited by small sample sizes, inconsistent protocols, and a lack of high-quality randomized controlled trials.

The rationale for combining WBV with intensive therapeutic exercise and functional training lies in their complementary mechanisms of action. Intensive therapy targets motor learning through task repetition and neuroplastic adaptation, while WBV may enhance these effects by increasing afferent input, facilitating voluntary muscle activation, and priming the central nervous system for improved motor responses. This synergy could theoretically amplify the therapeutic effects, leading to greater gains in strength, mobility, and functional independence. Despite the non-invasive nature and adaptability of WBV, its integration into standard CP rehabilitation remains limited due to insufficient robust evidence when compared to well-established physiotherapy programs [12,13,14,15].

This clinical trial aims to determine whether incorporating WBV into an intensive therapeutic exercise and functional training program yields statistically significant improvements in children with CP. Specifically, the study will compare short-, medium-, and long-term effects on lower-limb motor function, spasticity, isometric muscle strength, and balance, ultimately providing robust evidence to support its clinical implementation.

## 2. Materials and Methods

### 2.1. Study Design

A randomized controlled trial was conducted between March and October 2024 at the Department of Physiotherapy and Rehabilitation of the Hospital Niño Jesús in Madrid, Spain. The study adhered to the guidelines of the CONsolidated Standards of Reporting Trials (CONSORT) 2010 statement [21] and the Enhancing the QUAlity and Transparency Of health Research (EQUATOR) network [22] to ensure methodological rigor and transparency in health research. The trial was registered at ClinicalTrials.gov (NCT06330311 and date of 19 March 2024). Ethical approval for the study protocol was obtained from the Ethics Committee of Hospital Clínico San Carlos (code 20/551-EC_X, date of approval 21 July 2020).

### 2.2. Participants

Participants were referred by hospital physicians and met the following inclusion criteria: a confirmed diagnosis of spastic cerebral palsy; GMFCS levels I, II, or III; the ability to stand and walk independently with or without assistive devices; age between 8 and 14 years; and sufficient comprehension and ability to follow instructions.

The exclusion criteria for the study were as follows: the presence of fixed contractures in the lower limbs; botulinum toxin injections or serial casting within the past three months; a history of orthopaedic surgery within the past 12 months; participation in muscle strengthening programs within the past four months; and any medical contraindications to intensive physical exercise.

### 2.3. Randomization and Masking

A total of 30 children were randomly assigned to either the experimental or control group (15 participants per group) using a randomization table specifically developed for this study by Moses and Oakson [23]. To ensure allocation concealment, individual, sequentially numbered cards indicating group assignment were placed in sealed, opaque envelopes and folded using the closed-envelope method. Randomization was performed by a researcher from Universidad Complutense de Madrid who was not involved in participant evaluation or intervention.

All interventions were administered by researchers from Universidad Complutense de Madrid, while baseline and post-intervention assessments at 1, 2, and 6 months were conducted by the principal investigator, who was blinded to both the intervention and the randomization process.

Participants were instructed to continue their usual treatments and medical check-ups throughout the study period.

### 2.4. Intervention

Both groups underwent a 4-week intervention consisting of four sessions per week. The intensive physiotherapy program incorporated goal-oriented activities and therapeutic exercises. The protocol included two 7 min treadmill walking sessions per session, with assistance as needed through body weight suspension or lower limb support, complemented by a muscle strengthening and lower limb coordination program.

The exercise regimen comprised wall squats using a fitball, forward and lateral lunges on a step, plantar flexor exercises integrated into play activities, stair climbing, single-leg balance tasks, and supine glute bridges. Load adjustments were implemented based on a 10% improvement criterion, as recommended by Van Vulpen et al. [24].

Exercises were performed at maximum possible speed. Each participant was instructed to complete the highest number of repetitions within 30 s, performing a total of four sets with a one-minute rest interval between sets.

The experimental group additionally received daily WBV therapy using a Galileo S 35 vibration platform (Novotech Medical GmbH, Pforzheim, Germany). This side-alternating platform operates within a frequency range of 5–33 Hz and provides an amplitude range from 0 to ±4.7 mm (peak-to-peak displacement up to 9.4 mm), depending on foot positioning on the platform.

Participants wore their usual footwear but did not use foot or ankle orthoses during the intervention. They were positioned on the vibration platform with their feet placed parallel to each other and maintained a slight flexion of the knees, ensuring a stable and symmetrical stance throughout the sessions.

The WBV parameters were limited to low frequencies (maximum frequency of 20 Hz) in accordance with the protocol proposed by Gusso et al. [25] and Pin et al. [26], who demonstrated the safety and efficacy of these settings in children and adolescents with CP classified within GMFCS levels I to III. The protocol was administered over a 4-week intervention period and consisted of three sets with a progressive increase in vibration amplitude and frequency (up to 20 Hz and 2 mm) to facilitate gradual adaptation to the intervention. The maximum vibration frequency reached was 20 Hz. Low-frequency vibration has been associated with a lower risk of adverse effects such as excessive muscle fatigue, joint overload, or discomfort, particularly in paediatric populations and with motor impairments. Considering that participants were concurrently engaged in an intensive therapeutic exercise and functional training program, it was deemed essential to avoid adding an intervention that could induce additional fatigue.

### 2.5. Data Collection

Four follow-up assessments were conducted: at baseline, immediately post-intervention, and at two and six months post-intervention.

Prior to the follow-up assessments, parents or legal guardians of potential participants who had expressed interest in the study were contacted by telephone. During this initial contact, an information sheet detailing the study was provided, and any questions were addressed. Eligibility was confirmed by verifying compliance with the inclusion criteria and the absence of exclusion criteria.

Upon confirmation of willingness to participate, families were scheduled for an in-person interview at the Physiotherapy Unit of the Departmental Section of Radiology, Rehabilitation, and Physiotherapy. During this meeting, the information sheet, informed consent form, and confidentiality/privacy agreement were provided. Once signed consent was obtained, the baseline assessment was initiated.

The primary outcome of this study was gross motor function, assessed using the Gross Motor Function Measure (GMFM-88). The validated Spanish version of the GMFM-88 scale consists of 88 items distributed across five dimensions, scored on a 4-point scale (0, 1, 2, 3) [27]. This study focused on dimensions D (standing) and E (walking, running, and jumping), where higher scores indicate better motor function.

Secondary outcome measures included lower limb muscle tone assessed with the Modified Ashworth Scale (MAS), isometric muscle strength of the lower limbs measured with a hand-held dynamometer, and balance evaluated using the Mini-BESTest.

The MAS is a clinical tool used to measure the increase in muscle tone during passive stretching on a 6-point scale (0-4) [28]. The assessment focused on the hip adductors and flexors, knee flexors and extensors, and the gastrocnemius and soleus muscles. In participants with hemiplegic patterns, data from the affected limb were considered for the analysis [29].

Isometric muscle strength of the lower limbs was assessed using the validated Handheld Dynamometer MicroFET^®^ 2 (Hoggan Health Industries, West Jordan, UT, USA). This device has a high threshold range from 1.4 kgf to 135 kgf, in increments of 0.5 kgf. The dynamometer was calibrated prior to the study according to the manufacturer’s specifications to ensure accuracy. Standardized positioning and testing procedures were followed based on protocols published by Crompton et al. [30] and Thorborg et al. [31], which are considered reliable for strength assessment in children with cerebral palsy. All measurements were conducted by the same trained physiotherapist to minimize inter-rater variability. Each muscle group was tested three times, and the mean value was used for analysis. The hip abductors, extensors, and flexors; knee extensors and flexors; and plantar and dorsal flexors of the ankle were assessed. For participants with hemiplegia, the affected limb was measured.

The Mini-BESTest is a balance assessment test that includes 14 items across 4 categories (anticipatory postural control, reactive postural control, sensory orientation, and dynamic gait). Scores range from 0 to 28, with higher scores indicating better balance performance [29].

### 2.6. Sample Size Calculation

The sample size was performed using the GRANMO v7.12 software [32]. The sample size calculation indicated that a total of 30 patients would be sufficient to detect statistically significant differences in the primary outcome, assuming an alpha level of 0.05 and accounting for an anticipated 10% loss to follow-up.

### 2.7. Statistical Analysis

All statistical analyses were conducted using the Jamovi statistical package version 2.4.12 (Sydney, Australia) [33], with a significance level set at *p* < 0.05 and 95% confidence intervals (CI). Data normality was verified prior to analysis, and descriptive statistics were used to summarize the data. Normally distributed data were presented as means, standard deviations (SD), and 95% CI. Baseline between-group comparability was assessed using a multivariate general linear model for continuous variables.

For the comparative analysis of each dependent variable, a mixed linear model was employed, considering subjects as a random factor. Fixed factors in the analysis included the group assignment (experimental vs. control) and the time points of evaluation (baseline, 1 month post-intervention, 2 months post-intervention, and 6 months post-intervention). This model allows the inclusion of all available data, regardless of whether participants completed the intervention, enabling analysis based on the full dataset, even if some participants were not assessed at the final time point.

## 3. Results

### 3.1. Descriptive Analysis

**Figure 1:**
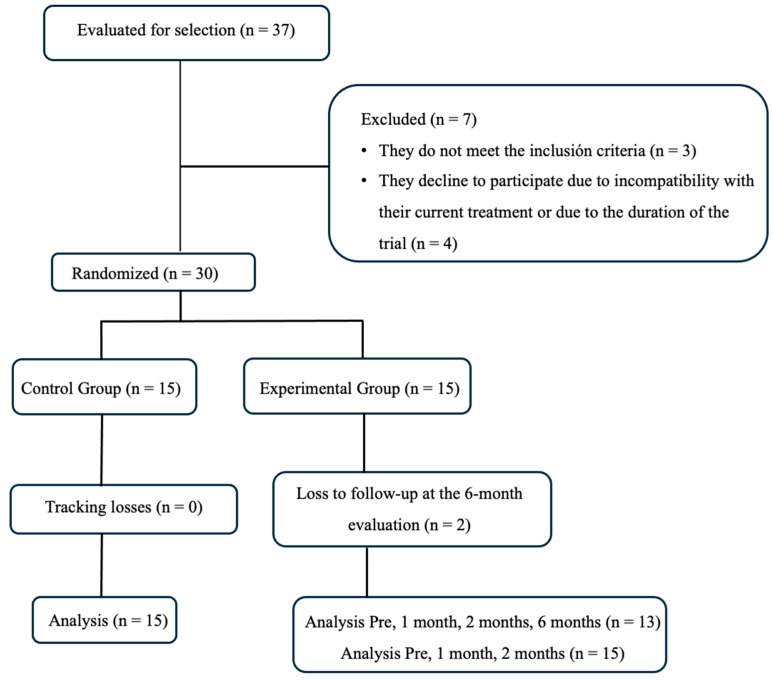
The flowchart illustrates the process of participant selection, randomization, allocation, and analysis in the two-study group.

Thirty-seven individuals were assessed for eligibility. Of these, seven were excluded: three of them did not meet the inclusion criteria, and four declined participation due to incompatibility with their current treatment or the duration of the trial. No additional reasons for exclusion were reported (Figure 1). All participants were recruited from Hospital Niño Jesús in Madrid.

Thirty participants were randomly assigned to an experimental group (EG, *n* = 15) and a control group (CG, *n* = 15). We found no differences between the groups in terms of clinical and sociodemographic characteristics (Table 1), nor baseline outcomes (Table 2). Both groups received the assigned intervention, with no interruptions in the intervention and no exclusions from the analysis. Only two participants from the EG were lost to follow-up at six months post-intervention.

### 3.2. Comparative Analysis

The results of the analysis of variance (ANOVA) for each study variable are presented below.

#### 3.2.1. Gross Motor Function

The results for the main variables in dimensions D and E of the GMFM showed significant improvements from baseline in both groups (*p* < 0.001), with no significant differences between the groups (Table 3) or across the study period (*p* = 0.09 and *p* = 0.86, respectively).

#### 3.2.2. Muscle Tone and Strength

We found a reduction in muscle tone and an increase in muscle strength from baseline to follow-up (Table 4). No differences were found between the groups, and no interaction was observed between the measurement time points and the intervention group. Although in the case of strength we found an interaction between the measurement time points and the muscle groups studied (*p* = 0.01), we observed a greater increase in muscle strength after treatment (1 month) of the hip extensors and plantar flexors.

#### 3.2.3. Balance Results

Participants’ balance improved in all balance dimensions from baseline to the follow-up (*p* < 0.001) (Table 5). No differences were found between the groups, and no interaction between the measurement time and intervention group was observed.

## 4. Discussion

The aim of this study was to assess the efficacy of WBV combined with intensive therapeutic exercise and functional training in children with CP. Both groups—control and experimental—showed improvements in functional capacity (GMFM-88), spasticity, muscle strength, gait, and balance. These improvements were maintained at follow-up, suggesting that the effects were largely attributable to the physiotherapy and functional training program. The addition of WBV did not result in statistically significant enhancements.

Although no statistically significant differences were found between the groups, the experimental group showed a consistent trend toward improvement in all outcome variables. These findings are in line with those reported by Pulay et al. [34], who concluded that WBV may have beneficial effects as an adjunct therapy in children with cerebral palsy, particularly when combined with conventional physiotherapy. While the addition of WBV did not result in statistically significant enhancements beyond those achieved through intensive physiotherapy alone, the observed trends suggest that WBV could offer additional clinical benefits and may be a useful complementary intervention in pediatric rehabilitation for children with CP.

However, our findings contrast with those of Cai et al. [19], whose meta-analysis reported significant improvements in GMFM-88 dimensions D and E, balance, mobility, and ankle range of motion with the addition of WBV [35,36,37,38]. These discrepancies may be partially explained by differences in intervention duration, vibration frequency, and intensity of baseline physiotherapy programs. Notably, several studies reporting significant effects used longer interventions (8–12 weeks), higher WBV frequencies (>25 Hz), and lower session frequencies per week, which may allow better neuromuscular adaptation and reduce the risk of fatigue [16,39,40,41].

Our study aimed to evaluate whether shortening the total intervention duration while maintaining the total number of sessions could be a viable therapeutic option, considering the challenges of conducting a long-term study with non-hospitalized children. This compressed schedule, coupled with a progressive increase in WBV intensity (10 to 20 Hz), following the protocol of Gusso et al. [25] and Pin et al. [26], may have limited the cumulative physiological impact of WBV. The most intensive vibration exposure began only after the first week, further shortening the effective treatment period. In contrast, most studies reporting significant WBV effects used frequencies exceeding 20 Hz, with some reaching up to 40 Hz, as seen in Katusic et al. [35]. This factor may have influenced our results, as the initial sessions involved lower WBV intensity, potentially limiting its impact.

The intensity and quality of the baseline physiotherapy protocol represent another key factor. In contrast to other studies that vaguely described the control intervention as “conventional physiotherapy”, our program was highly structured and intensive, potentially minimizing the relative contribution of WBV. These differences in intervention design should be considered when interpreting outcomes. In the few studies that describe the intervention, it follows a “wait and see” approach. This lack of clarity in defining “usual care” has been highlighted in a recent systematic review by Arienti et al., who emphasized the variability and often insufficient reporting of control interventions in stroke rehabilitation trials, describing it as a “black hole” in clinical research [42].

Overall, our study contributes to the ongoing debate about the role of WBV in pediatric neurorehabilitation. Although the present study did not show statistically significant improvements with the addition of WBV to intensive therapy, the intervention was safe, well tolerated, and demonstrated a favorable trend. These findings align with previous studies and suggest that WBV may be considered as a complementary option in pediatric neurorehabilitation.

Further high-quality research with larger samples, standardized protocols, and extended follow-up is warranted to determine the full extent and benefits of WBV for children with cerebral palsy.

### Study Limitations

Several limitations should be considered in the present study. The small sample size (*n* = 30) is a major limitation, affecting both the statistical power to detect significant differences and the feasibility of conducting subgroup analyses. Although subgroup analyses based on GMFCS levels could offer valuable insights into the differential effects of the intervention, the small sample size and uneven distribution across functional levels—particularly the low number of participants classified as GMFCS Level III—limited the feasibility and statistical reliability of such analyses. This remains an important consideration for future studies with larger and more balanced samples.

The heterogeneity of the sample, in terms of both topographic distribution (e.g., hemiparesis, diplegia) and functional levels (GMFCS I–III), may have influenced the variability of responses to the intervention and potentially diluted observable group effects. While this diversity reflects real-world clinical populations, it also represents a methodological limitation that could mask treatment-specific outcomes. Future studies with more homogeneous or stratified samples may better elucidate the specific effects of WBV.

In addition, the lack of blinding for therapists and participants may have introduced bias. Although blinding was not feasible due to the nature of the intervention, we acknowledge that both therapist expectations and parent/participant awareness of treatment allocation could have influenced outcomes. This limitation is common in rehabilitation studies but should be carefully considered when interpreting the results.

Regarding the intervention protocol, the frequency used in the WBV therapy was 20 Hz, which is lower than the 25–40 Hz range used in many other studies. This could have influenced the magnitude of the observed effects. In addition, the control group did not receive placebo WBV, which limited both participant and practitioner blinding. The physiotherapists, as well as parents and legal guardians, were aware of the group assignments, and many children preferred to be accompanied by family members during the sessions.

Another relevant aspect is that all participants continued their routine physiotherapy treatments during the study period. Although this reflects a real-world clinical setting, it may have masked the isolated effects of WBV. Furthermore, the short-term, intensive application of the protocol may have contributed to overall fatigue, potentially masking any additive effect of WBV. Muscle fatigue, not measured in our study, could also have diminished performance during sessions.

## 5. Conclusions

This study demonstrates that both an intensive therapeutic exercise and functional training program, whether alone or combined with a whole-body vibration intervention, lead to improvements in motor functional capacity in children with cerebral palsy, with no significant differences between the two treatment approaches.

The significant improvements observed within the groups are likely attributable to the intensive physiotherapy treatment, which included therapeutic exercises and functional training. This confirms the importance of such physiotherapy interventions in enhancing the functional capacity of children with spastic cerebral palsy.

Further research on whole-body vibration is needed to determine whether specific vibration parameters (duration, frequency, intensity), physiotherapy protocols, or session timing could optimize these outcomes.

## Figures and Tables

**Table 1:** Clinical characteristics of the participants.

Variables		CG	EG	T; *x*^2^	*p*
**Age**	Mean ± SD	11.2 ± 2.0	10.5 ± 1.7	1.08	0.29
**Sex**	Male Female	6 (40%) 9 (60%)	8 (53.3%) 7 (46.7%)	0.54	0.46
**Topographical** **pattern**	Deprecia Right Hemiparesia Left Hemiparesia Tetraparesia	8 (26.7%) 2 (6.7%) 4 (13.3%) 0 (0%)	8 (26.7%) 3 (10%) 2 (6.7%) 1(3.3%)	3.74	0.73
**GMFS**	I II III	4 (13.3%) 10 (33.3%) 1 (3.3%)	10 (33.3%) 4 (13.3%) 1 (3.3%)	5.14	0.08

SD: standard deviation; EG: experimental group; CG: control group.

**Table 2:** Baseline outcome scores.

Variables		Mean ± SD		Student’s T		Levene	
		CG	EG	T	*p*	F	*p*
Gross Motor GMFM88	Dimension D	32.7 ± 4.1	33.7 ± 3.3	−0.73	0.47	0.22	0.64
	Dimension E	60.5 ± 10.4	60.5 ± 9.0	0.02	0.98	0.03	0.86
Muscle tone (MAS)	Hip Adductors	0.6 ± 0.6	0.4 ± 0.5	0.98	0.34	1.25	0.27
	Hip Flexors	0.3 ± 0.4	0.3 ± 0.5	0.63	0.53	0.02	0.88
	Knee Flexors	1.1 ± 0.6	1.1 ± 0.6	0.08	0.94	0.23	0.64
	Knee Extensors	0.5 ± 0.6	0.5 ± 0.80	−0.13	0.90	0.05	0.83
	PF-Soleus	1.1 ± 0.5	1.1 ± 0.3	−0.53	0.60	0.69	0.41
	PF-Gastrocnemius	1.4 ± 0.3	1.7 ± 0.4	−0.13	0.90	0.75	0.39
Muscle Strength (Handheld dynamometry)	Hip Abductors	9.8 ± 3.3	11.0 ± 3.3	−0.98	0.34	0.15	0.70
	Hip Flexors	11.1 ± 3.1	12.1 ± 2.6	−0.90	0.38	0.44	0.51
	Hip Extensor	8.1 ± 3.7	10.5 ± 4.3	−1.65	0.11	0.89	0.35
	Knee Flexors	7.6 ± 2.6	8.1 ± 3.1	−0.47	0.65	0.67	0.42
	Knee Extensors	12.7 ± 5.3	13.4 ± 2.8	−0.46	0.65	8.80	0.01
	Dorsal Flexors	6.1 ± 2.5	6.4 ± 1.4	−0.14	0.90	4.46	0.04
	Plantar Flexors	14.5 ± 4.6	15.1 ± 3.8	−0.39	0.70	1.74	0.20
Balance (Mini-BESTest)	Anticipatory	3.7 ± 1.0	4.1 ± 0.7	−0.78	0.44	0.33	0.57
	Reactive PC	3.8 ± 1.5	3.6 ± 1.8	0.46	0.65	1.88	0.18
	Sensory Orientation	4.3 ± 1.6	4.9 ± 1.4	−1.01	0.32	2.87	0.10
	Dynamic Gait	7.5 ± 1.5	8.0 ± 1.1	−1.00	0.33	0.99	0.33

SD: standard deviation; CG: control group; EG: experimental group; MAS: Modified Ashworth Scale; PF: plantar flexor; PC: postural control.

**Table 3:** Comparative analysis of the GMFM-88 scale assessment in dimensions D and E.

GMFM 88		Pre	1 Month	2 Months	6 Months	Moment (M)		Group (G)		Mx × G	
		Mean ± SD	Mean ± SD	Mean ± SD	Mean ± SD	F	*p*	F	*p*	F	*p*
**D**	CG	32.7 ± 4.1	34.9 ± 3.2	34.9 ± 3.0	34.5 ± 3.7	26.73	<0.001	0.07	0.80	22.69	0.09
	EG	33.7 ± 3.4	35.2 ± 3.1	34.8 ± 3.1	34.2 ± 3.4						
**E**	CG	60.5 ± 10.4	63.8 ± 9.2	63.6 ± 10.0	62.9 ± 10.0	35.54	<0.001	0.01	0.91	0.25	0.86
	EG	60.5 ± 9.2	64.3 ± 7.0	64.0 ± 6.8	62.6 ± 7.8						

SD: standard deviation; D: dimension D; E: dimension E; CG: control group; EG: experimental group.

**Table 4:** Comparative analysis of the muscle tone (MAS) and strength (handheld dynamometry).

	Muscle Tone (MAS)		Muscle Strength (Handheld Dynamometry)	
	F	*p*	F	*p*
Moment	28.13	<0.001	0.37	<0.001
Group	0.06	0.80	98.18	0.55
Muscle	203.12	<0.001	245.99	<0.001
Moment × Group	0.18	0.91	1.48	0.22
Moment × Muscle	1.02	0.44	2.03	0.01
Group × Muscle	1.43	0.21	1.25	0.28
Group × Moment × Muscle	0.27	0.99	0.37	0.99

MAS: Modified Ashworth Scale.

**Table 5:** Comparative analysis of balance (MiniBESTest).

	Anticipatory		Reactive PC		Sensory Orientation		Dynamic Gait	
	F	*p*	F	*p*	F	*p*	F	*p*
Moment	20.50	<0.001	37.96	<0.001	8.83	<0.001	14.01	<0.001
Group	0.69	0.41	0.01	0.94	1.42	0.24	0.47	0.49
Moment × Group	0.95	0.42	0.75	0.52	0.29	0.83	0.70	0.55

PC: postural control.

## Data Availability

The data that support the findings of this study are available from the corresponding author, upon reasonable request.

## Abbreviations

The following abbreviations are used in this manuscript:

CG	Control group
CI	Confidence interval
CP	Cerebral palsy
EG	Experimental group
GMFCS	Gross Motor Function Classification System
GMFM	Gross Motor Function Measure
kgf	Kilogram force
MAS	Modified Ashworth Scale
SD	Standard deviation
WBV	Whole-body vibration
